# Performance of Kala-Azar Surveillance in Gaffargaon Subdistrict of Mymensingh, Bangladesh

**DOI:** 10.1371/journal.pntd.0003531

**Published:** 2015-04-10

**Authors:** Kazi Mizanur Rahman, Indira V. M. Samarawickrema, David Harley, Anna Olsen, Colin D. Butler, Shariful Amin Sumon, Subrata Kumar Biswas, Stephen P. Luby, Adrian C. Sleigh

**Affiliations:** 1 International Centre for Diarrhoeal Disease Research, Bangladesh, Dhaka, Bangladesh; 2 National Centre for Epidemiology and Population Health, Research School of Population Health, The Australian National University, Canberra, Australian Capital Territory, Australia; 3 Population and Social Health Research Program, Griffith Health Institute, Gold Coast Campus, Griffith University, Gold Coast, Queensland, Australia; 4 University of New South Wales, Sydney, New South Wales, Australia; 5 University of Canberra, Canberra, Australian Capital Territory, Australia; Emory University, UNITED STATES

## Abstract

**Introduction:**

Elimination of kala-azar is planned for South Asia requiring good surveillance along with other strategies. We assessed surveillance in Gaffargaon *upazila* (a subdistrict of 13 unions) of Mymensingh district, Bangladesh highly endemic for kala-azar.

**Methods:**

In 4703 randomly sampled households, within nine randomly sampled villages, drawn from three randomly sampled unions, we actively searched for kala-azar cases that had occurred between January 2010 and December 2011. We then searched for medical records of these cases in the patient registers of Gaffargaon *upazila* health complex (UHC). We investigated factors associated with the medical recording by interviewing the cases and their families. We also did a general observation of UHC recording systems and interviewed health staff responsible for the monthly reports of kala-azar cases.

**Results:**

Our active case finding detected 58 cases, but 29 were not recorded in the Gaffargaon UHC. Thus, only 50% (95% CI: 37%–63%) of kala-azar cases were reported via the government passive surveillance system. Interviews with health staff based in the study UHC revealed the heavy reporting burden for multiple diseases, variation in staff experience, high demands on the staff time and considerable complexity in the recording system. After adjusting for kala-azar treatment drug, recording was found more likely for those aged 18 years or more, males, receiving supply and administration of drug at the UHC, and more recent treatment.

**Discussion:**

Fifty percent of kala-azar cases occurring in one highly endemic area of Bangladesh were recorded in registers that were the source for monthly reports to the national surveillance system. Recording was influenced by patient, treatment, staff and system factors. Our findings have policy implications for the national surveillance system. Future studies involving larger samples and including interviews with health authorities at more central level and surveillance experts at the national level will generate more precise and representative evidence on the performance of kala-azar surveillance in Bangladesh.

## Author Summary

Visceral leishmaniasis, a parasitic disease transmitted by sandflies, is known as kala-azar in South Asia, and has been targeted for elimination in that region. The aim is to reduce its incidence to a low level so that it is no longer a public health problem. Elimination strategies include good surveillance for occurrence of the disease. We assessed surveillance in Gaffargaon subdistrict of Mymensingh district in Bangladesh where occurrence of kala-azar is high. We randomly sampled 4703 households and searched for kala-azar cases that had occurred between January 2010 and December 2011. We then searched for medical records of these cases in the patient registers of Gaffargaon *upazila* or subdistrict health complex (UHC). We interviewed cases, their families and health staff responsible for the monthly reports of kala-azar. We also observed the UHC recording system. We detected 58 cases, but 29 (50%) were not recorded in the Gaffargaon UHC. Problems include the heavy reporting burden for multiple diseases, variation in staff experience, high demands on the staff time and complexity in the recording system. Recording was more likely for adults, males, those given drugs at the UHC, and more recent treatment. Our findings have implications for kala-azar surveillance.

## Introduction

Visceral leishmaniasis (VL), caused by protozoan parasites transmitted by sandflies, is a systemic illness characterized by fever and splenomegaly. The disease is endemic in impoverished tropical areas globally [1–3], and in South Asia is known as kala-azar (black-fever) and only affects humans.

The governments of India, Bangladesh and Nepal in 2005 committed to eliminate kala-azar by 2015. Elimination is defined as annual incidence of less than 1 per 10,000 population at the district or sub-district level [4]. Surveillance is one of the main strategies for kala-azar elimination [5]. In Bangladesh, surveillance provides estimates and trends of the nationwide occurrence of kala-azar [6]. Kala-azar is reported every month starting from the *upazila* or subdistrict level. We have studied surveillance in a highly endemic sub-district of Bangladesh and here we report our findings. We analyse factors influencing case recording and make recommendations to improve surveillance.

## Methods

### Study setting

The study was conducted in Gaffargaon sub-district of Mymensingh district in Bangladesh, 80km north-east of Dhaka. Gaffargaon is divided into 15 unions and 214 villages. Around half of the population of Gaffargaon are male, half are literate and most are farmers. The sub-district, 398 sq. km. with a population of 430,000, reported the third highest number of kala-azar cases in 2011[7,8].

### Study sample and case detection

We expected 1.4 kala-azar cases per 100 households (based on an unpublished 2009 survey data). Also, using Epi Info StatCalc, we calculated that we would need a sample of 68 cases (i.e. 4857 households) to detect 50% under-reporting through the national surveillance system with 10% precision and 90% confidence [9]. First, we excluded two of the 15 unions that were part of central administration. Care seeking experience of kala-azar patients from these two unions would be different than that of the patients from the other unions because of the proximity to the Gaffargaon *upazila* health complex (UHC). Then we randomly sampled three unions from the sampling frame of 13 unions. In each of these sampled unions we randomly selected three villages. We then sampled households in each village, aiming for about 600 households per village. If a village had more than 600 households we divided it into different paras or localities and randomly sampled paras until we got 600 households. If a village had less than 600 households, we sampled all the households of that village. This resulted in sampling of less number of households than expected. The resulting sample included a total of 4703 households from nine sampled villages from three sampled unions of Gaffargaon.

Our case definition required diagnosis by a qualified health care provider based on clinical presentation and a positive confirmatory diagnostic test. Most of the study respondents could not mention the name of the confirmatory diagnostic test. But they mentioned that the providers who confirmed kala-azar did so based on positive diagnostic test results for kala-azar. Going from sampled house to house in the period December 2011 to May 2012, we sought cases that had occurred between January 2010 and December 2011. In each household the informant was a case or a senior family member.

### Data collection: Interviews, observations and UHC records

If there was more than one kala-azar case in a household, we studied the earliest case in order to avoid household clustering. Kala-azar cases and their families were interviewed using a structured questionnaire. For cases less than 18 years of age, a parent or carer was interviewed. Surviving family members of deceased cases were interviewed. We collected demographic and economic data, date of symptom onset, signs and symptoms, care seeking experiences and diagnosis.

We also studied recording and reporting of kala-azar cases making general observations at the Gaffargaon UHC. As well, we interviewed three key health service staff from the Gaffargaon UHC who were involved in the kala-azar reporting system. We asked them to detail their role in kala-azar reporting. We asked about obstacles to reporting and how the reporting system could be improved. These semi-structured interviews were audio-recorded.

We examined Gaffargaon UHC records to determine if cases we detected had been entered in the registers. Inpatient, outpatient and laboratory registers were reviewed. We sought record entries in the 2010–2011 periods for all kala-azar cases we detected in the community. We used the name and address to confirm identification of the patients in the register books.

### Data management and analysis

Quantitative data were digitized using Epi Info (version 3.5.3). Information from the patients and families was recorded in MS Excel in a file which also noted whether the patient was recorded in the UHC registers. Qualitative data included extensive notes on general observations. The recordings were transcribed in the Bengali language for the three UHC staff.

Quantitative data were analysed using STATA version 8 [10]. We compared frequencies of patient and health system factors for those found and not found in hospital records. The factors were age, sex, place of diagnosis, treatment location, drug and year. Any difference with p<0.05 was considered statistically significant.

Interview transcripts were contrasted for recurring themes and informative quotations related to the research questions. Quotations used in this publication were translated into English by author KMR. All analyses were supported by the general observations.

### Ethics

Informed written consent was obtained from participants. For illiterate interviewees, the consent form was read out loud and the participants’ fingerprints were obtained on the consent forms. Before conducting the study of the kala-azar reporting system and interviewing staff of Gaffargaon UHC, written permission was obtained from the relevant authority based centrally in Dhaka and at Gaffargaon. Individual consent was also obtained from interviewed staff who were advised that they would not be identified. Ethical approvals were provided by the Human Research Ethics Committee of the Australian National University and the Ethical Review Committee of the International Centre for Diarrhoeal Disease Research, Bangladesh (icddr,b). Both the ethics committees specifically approved the use of thumb print procedures.

## Results

### Study participants

We screened 4703 households and identified 89 people fulfilling our case definition of kala-azar. Before further analysis, we excluded cases whose circumstances did not represent the typical probability for notification from Gaffargaon UHC. These exclusions included those who migrated out (5 cases) and those who received special clinical care or care from outside Gaffargaon (9 cases). As well, to avoid household clustering, we excluded 15 cases that arose in a family that had already provided a case for our sample. Two other cases refused to participate so 58 remained for analysis in our study of surveillance.

### Kala-azar patient flow and monthly reporting

**Fig 1:**
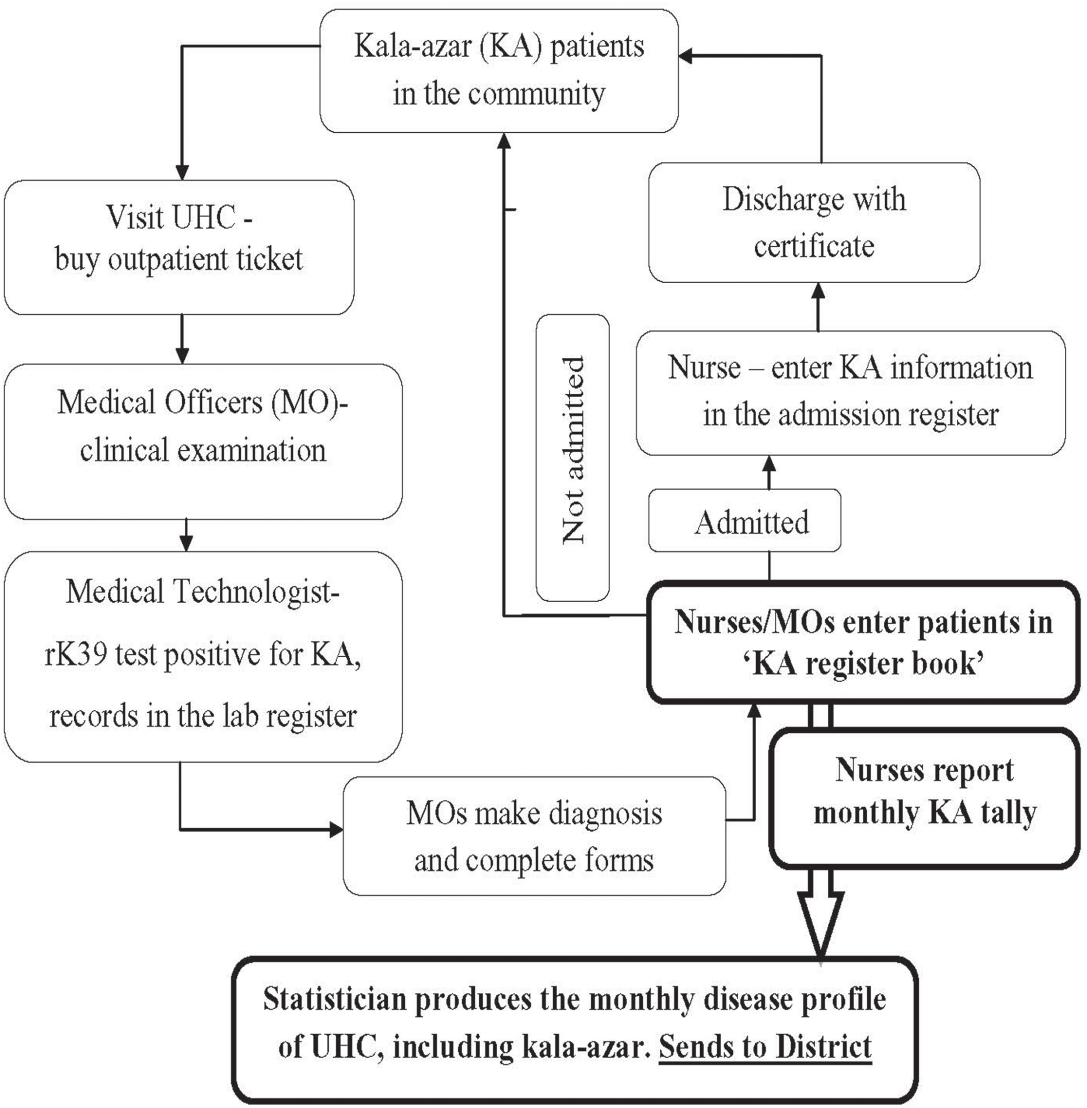
KA patient flow and reporting system observed in Gaffargaon UHC, 2012. Note: KA = kala-azar; UHC = *upazila* health complex; MO = medical officer.

The flow of kala-azar patients and related information is shown in Fig 1. We identified three registers (laboratory, kala-azar and admission) where staff recorded the patient name and other details. Each month, using the kala-azar register as the final source of the information, the responsible nurse reports to the UHC statistician the consolidated data on the number of kala-azar cases (by age, sex, treatment status). Then the statistician compiles a monthly kala-azar tally along with the routine ‘disease profile’ report and sends this to the Civil Surgeon’s office responsible for that district.

### Surveillance of kala-azar and factors influencing reporting

After reviewing the UHC registers in Gaffargaon we concluded that there were no records for 29 of our 58 actively detected cases. Thus, overall, 50% (95% CI: 37%–63%) were recorded by the government surveillance system—44% in 2010 and 59% in 2011. Accordingly we can estimate that no more than 50% (95% CI: 37%–63%) of kala-azar cases were reportable through the government mechanism that depends on UHC recording.

Using the survey data we explored factors associated with recording in the UHC kala-azar register. Patient attributes showed some associations as follows: age 18 years or more and male sex increased the likelihood of being recorded by the UHC, although none of the associations were statistically significant (Table 1).

**Table 1:** Factors influencing UHC recording of kala-azar patients.

Factor	Kala-azar cases detected		Odds ratio* # (95% CI)
	Recorded by UHC [N = 29], n (%)	Not recorded by UHC [N = 29], n (%)	
**Age**			
< 18 years	16 (55)	21 (72)	referent
18 years or more	13 (45)	8 (28)	3.3 (0.7–10.0)
**Sex**			
Female	8 (28)	12 (41)	referent
Male	21 (72)	17 (59)	2.2 (0.6–8.1)
**Facility/ provider confirmed kala-azar for the first time**			
Private	18 (62)	15 (52)	referent
Public	11 (38)	14 (48)	0.5 (0.1–2.0)
**Location KA drug administered**			
Not at UHC	24 (83)	13 (45)	referent
At UHC	5 (17)	16 (55)	1.8 (0.3–11.5)
**Year of KA treatment**			
2010	16 (55)	20 (69)	referent
2011	13 (45)	9 (31)	1.9 (0.6–6.5)

*Relative odds of being recorded in the UHC registers

#Odds ratio after adjusting for kala-azar treatment drugs (SAG or miltefosine) because miltefosine is only available through the UHC and thus more likely to associate with recording [16]

We also found certain health system factors associated with recording in the UHC kala-azar register. First, place of diagnosis (public vs. private) was indicative (OR = 0.5; 95% CI = 0.1–2.0). As well, place of drug supply was indicative: among those recorded 100% were supplied their treatment at the UHC as compared to 76% of those who were not recorded (p <0.01). Actual administration of kala-azar drug at the UHC was associated with 80% higher odds of being recorded (OR = 1.8; 95% CI = 0.3–11.5). We also observed that the probability of being found in the UHC record further improved in 2011 relative to 2010 (OR = 1.9; 95% CI = 0.6–6.5).

Interviews with staff indicated a number of factors influencing reporting. There were issues such as the burden of recording numerous notifiable diseases on the monthly ‘disease profile’ report.

“It is not just one report that we need to prepare from the hospital every month … All diarrhoea, ARI, kala-azar (cases) …(plus)… reports from the field (administrative level lower than the subdistrict), we send all these consolidated figures.”

As well, the reporting system itself has changed, leading to new routines along with a new kala-azar reporting form.

“We used to send the report on kala-azar with other diseases in the monthly ‘disease profile’. In that form there was a row for kala-azar. Later a separate form for kala-azar was introduced. We now send reports on kala-azar both through the ‘disease profile’ as well as in the separate form.”

The informant indicated that reporting channels were sometimes complex. This reflected the need to get the information through quickly but this can be difficult in hard copy due to the need for a signature by the *Upazila* Health and Family Planning Officer (otherwise known as the Thana Health Administrator or THA).

“We send the reports to the office of Civil Surgeon. We often send them through email. Sometimes we send the numbers over telephone if there is any hurry. Even through mobile phone. …We send hard copies (later) after getting it signed by the THA.”

In addition, different nursing staff recorded the patient data at different times. This may cause the quality or completeness of recording to vary. However, the system used was unlikely to lead to duplicate recording because of the unique registration number.

“We put a serial number… this serial number is the registration number. … We show the patient only once.”

## Discussion

Only half of the kala-azar cases arising in Gaffargaon sub-district, a highly endemic area within Mymensingh district, were recorded in the *upazila* health complex records. Consequently the monthly tallies of kala-azar cases reported to the government of Bangladesh represent only about half of the actual cases that occur. Investigation of socio-demographic factors associated with non-recording was generally uninformative—all factors tested were not significantly associated and effect estimates had wide confidence limits revealing limited statistical power. However, health system factors had more influence. Recording in the UHC kala-azar register significantly associated with supply of drug (UHC) and place of administration of the drug (UHC). We observed improvement of recording in 2011 compared to 2010.

Previous population based studies in Bangladesh have not reported surveillance performance in any way comparable to our study [2, 11–13]. In India, Singh et al (2006) assessed kala-azar surveillance in Bihar in 2003. They searched households for people with fever for over 15 days duration and confirmed kala-azar with microscopic parasite identification in spleen or bone marrow aspirates. Sixty-five cases were detected through their active case search and only 8 (12.3%) were reported [14]. The same group in a later study found that only 17% of the cases were reported [15]. Those over 30 years of age were significantly less likely to be reported, but no other patient, family or health system factor was shown to be indicative.

Our study was done on a two-year sample of kala-azar patients in a geo-demographically defined segment of the Bangladesh population in a highly endemic area. We achieved our principal aim of estimating with reasonable accuracy the proportion of incident kala-azar cases being recorded by the health system (i.e. 50%). But our sample size did not have statistical power to enable conclusive sub-analyses of factors related to surveillance. However, the qualitative data we collected from the staff based at the study UHC were able to provide some perspective on the issue of preparing tallies and reporting to the government. The system in use involves recording in a kala-azar register which is the source for reporting kala-azar to the statistician to enable the monthly tally. The staff we interviewed made comments about the data flow and forms, and the figure we produced showed a rather complex system that could be simplified.

We were not able to determine the actual proportion reported because we could not separate the contribution expected from our sample from the contribution expected from the rest of the population served by the same UHC. If we could have searched for cases in the entire population of Gaffargaon (around 430,000) for a particular month then we would be able to compare the number of cases detected in the community and number of cases reported from the UHC. But this would require substantial resources.

We learnt from our study that around 50% of kala-azar cases in a highly endemic area of Bangladesh are not yet detectable by the government passive surveillance system. Obstacles to reporting were related to the reporting system and the reporting burden for multiple diseases. Future studies involving larger samples and including interviews with health authorities at more central level and surveillance experts at the national level will generate more precise and representative evidence on the performance of kala-azar surveillance in Bangladesh.

## Supporting Information

S1 Checklist STROBE checklist. (DOC)
